# Case-Histories in Pyorrhea Alveolaris

**Published:** 1914-06

**Authors:** Wm. Crenshaw

**Affiliations:** Atlanta, Ga.; Dean Atlanta Dental College


### CASE-HISTORIES IN PYORRHEA ALVEOLARIS.

By Wm. Crenshaw. D. D. S., Dean Atlanta Dental College.

In view of the wide-spread awakening of interest on the part of the medical profession in the relation of septic teeth and gums to systemic disorders, particularly rheumatism, indocarditis, and septic fevers, the writer feels that physicians generally will be interested in the sub-joined series of case-histories.

The list of cases could be run into the hundreds; but the number reported is sufficient to show what may be expected in the treatment of cases of advanced type.

Case No. 1. Mrs. P. H., age 35, first examined January 1913, complained of debility and lassitude, lack of appetite, and presented the usual pyorrhea fetor on the breath. This patient was under treatment for nasal catarrh, and was suffering from intestinal indigestion. In this case one of the pyorrhea pockets penetrated between the palatal and buccal roots, of an upper right molar and the maxillary sinus, and had set up a persistent inflammatory action there. The case yielded promptly to treatment with a general improvement in health, and concurrently with the healing of the pyorrhea the naasl trouble disappeared, which was caused by the penetration of the pyorrhea pocket into the sinus. Estimating as well as I can, I should say that the sum of the area of ulcerated surfaces in this mouth was not less than two and a half square inches; yielding probably six or eight drops of pus a day, which, due partly to the fact that pus has neither taste nor odor in the mouth to the patient, was swallowed with the food. This mouth was restored to a healthy condition and remains so.

Case No. 2. Miss F. H., age 20, came for examination in June, 1913, with ten teeth affected with pyorrhea. Two molars were beyond the promise of redemption. The upper central incisors with pockets at the central surfaces had migrated one half inch apart; and others of the affected teeth had changed position in both jaws to the extent it was necessary to employ an orthodontist to bring the two arches in proper occlusion. The area of ulceration in this case was about one square inch. The case yielded successfully to treatment.

Case No. 3. Dr. N. F. M., age 38, practicing physician, presented himself November 1913, with fifteen pyorrhetic ulcers. He gave evidence of septic poisoning. Intestinal indigestion, depression, lassitude, loss of appetite, and fever were concomitants in his case. This patient had taken treatment at a sanitorium of national reputation without the medical staff detecting the cause of his trouble. Upon discovery of the cause, he came to the writer, and had the pyorrhea treated, which yielded promptly, and which resulted in a return of health. This patient must have absorbed ten to twelve drops of pus daily; and the area of ulceration would have approximated two and a half square inches.

Case No. 4. Rev. Dr. P., age 50 presented with twenty-six teeth affected. Three of the number were removed, because beyond redemption. A pyorrhea pocket between the upper right first and second molar penetrated the maxillary sinus. This case had proceeded to a point of profound toxemia, which caused a general lassitude and breaking down of the physical forces. Within two months after the treatment was concluded, which consisted first of the removal of the deposits on the roots, and the curetting of the diesased tissues, the patient was restored to his usual health. The sum of the ulcerative surfaces in this case must have been two square inches.

Case No. 5. Mr. J. G. B. E., attorney, age 45, when first seen had eighteen teeth affected; of these two were removed, which were too far advanced to be treated. Pyorrheal migration had gone on with several of the teeth affected. Excess of pus exudation, with septic poisoning, languor and lowered vitality were symptoms when the patient came for treatment. The pockets were syringed out with an antiseptic wash, cocainized, and the surgical work done. Follow-up treatments for three weeks were made before the patient was dismissed. The case yielded, and the teeth were restored to normal use. The aggregate of ulcerative surfaces in this case was about two and a half square inches.

Case No. 6. Mr. R. L. M., attorney, aged 40, presented with twenty-eight teeth affected. The pockets were unusually full of pus, the pyorrhea fetor distinct and disagreeable. The pockets were syringed out and cocainized, and the surgical work done with prompt relief and restoration. This case must have exuded several drops of pus daily. Probalby three inches of ulcerated surface existed in this mouth.

Case No. 7. Miss J. H., age 21, presented with twenty affected teeth. The front lower teeth which were loose from the pockets around the roots were splinted with gold inlay and then operated on. Pus discharged from practically all the teeth. The case yielded. The sum of the ulcerated areas about two square inches.

Case No. 8. Mr. W. M. H., age 42, upon examination showed twelve teeth affected. This case had none of the complications met with ordinarily, and yielded promptly. There was less of pus exudation, and consequently less of ulcerative surfaces than in the majority of cases.

Case No. 9. Mr. J. E. B., aged 35, presented himself with twenty teeth affected. Pus discharged from practically all of them. The first molars below had pockets tunneling under the teeth through the bifurcation of the roots. The patient was anemic, weak and feeble; three splints were employed; but the case has not yielded satisfactorily.

Case No. 10. Mrs. Lem G., aged 35, twenty teeth affected, pus discharge, teeth sound and white. Case yielded in reasonable time.

Case No. 11. Mrs. F. M. G., aged 55, twenty-four teeth affected, pus discharge, and pyorrhea fetor. Ligament on palatal root of upper left molar entirely destroyed, and the nerve severed at point of the root, producing neuralgia over that side of the face, which had existed for the past twelve months. The nerve was removed, the root canals filled, after which the palatal root was amputated. The tooth remains in position on the two buccal roots and became firm and serviceable, neuraliga disappeared on the removal of the nerve of the affected tooth. There were probably two inches of ulcerated surface. Case yielded in three weeks' time.

Case No. 12. Capt. S. S., aged 55, presented with twelve teeth affected. Splinting of the lower incisors was necessary before the operation of curetting the pockets. The left upper third molar developed a pocket penetrating into the maxillary sinus. Treatment relieved the nasal derangement, and the teeth affected with pyorrhea.

Case No. 13. Mrs. F. F. F., aged 40, was treated while a patient of the Davis & Fischer Sanitorium, Atlanta. This case had twenty-two teeth affected with considerable pus exudation, and was of three or four years standing. The pyorrhea pockets were curetted and the calculus concretions removed from the teeth, with such follow-up treatment as is usual in these cases. This patient was treated while propped up in bed. Within three weeks the diesase had abated and the patient was able to sit up and take her food with relish, and to return home.

The following three cases show the danger and often fatal consequences of allowing pyorrhetic infection to go untreated too long. All three of these patients could have been saved by timely relief of the septic condition of their mouths.

Case No. 14. Mrs. S. H. W., aged 45, came with twenty teeth affected, was in low state of health. This case was of eight years' standing, and no satisfactory results were gotten. Patient died.

Case No. 15. Mr. C. H., aged 45, had affected, the full complement of 32 teeth. There was hardly less than four square inches of ulcerated pus exuding pockets. The pyorrhea in this case was of ten years' standing, and the writer was called in to the case by the attending physician, after systemic infection had made such headway as to cause endocarditis and create an unabating fever. The patient could not leave his room, nor sit up except for short periods. The pockets were curetted, but nothing of a satisfactory nature could be done in the advanced condition of this case and the patient died two months later.

Case No. 16. Mr. W. C. S., aged 36, was seen at his home, propped up in bed. He was given such treatment as could be administered under the circumstances. Practically all of the teeth were affected with pyorrhea of eight years' standing, and were exuding pus. Infection had advanced to the point of weakening the heart. The patient died after three months' confinement to bed.

Grant Bldg., 619-21.

